# Applying dynamic Bayesian networks to perturbed gene expression data

**DOI:** 10.1186/1471-2105-7-249

**Published:** 2006-05-08

**Authors:** Norbert Dojer, Anna Gambin, Andrzej Mizera, Bartek Wilczyński, Jerzy Tiuryn

**Affiliations:** 1 Institute of Informatics, Warsaw University, Banacha 2, 02-097 Warszawa, Poland; 2 Institute of Fundamental Technological Research, Polish Academy of Sciences, Świętokrzyska 21, 00-049 Warszawa, Poland; 3 Institute of Mathematics, Polish Academy of Sciences, Śniadeckich 8, 00-956 Warszawa, Poland

## Abstract

**Background:**

A central goal of molecular biology is to understand the regulatory mechanisms of gene transcription and protein synthesis. Because of their solid basis in statistics, allowing to deal with the stochastic aspects of gene expressions and noisy measurements in a natural way, Bayesian networks appear attractive in the field of inferring gene interactions structure from microarray experiments data. However, the basic formalism has some disadvantages, e.g. it is sometimes hard to distinguish between the origin and the target of an interaction. Two kinds of microarray experiments yield data particularly rich in information regarding the direction of interactions: time series and perturbation experiments. In order to correctly handle them, the basic formalism must be modified. For example, dynamic Bayesian networks (DBN) apply to time series microarray data. To our knowledge the DBN technique has not been applied in the context of perturbation experiments.

**Results:**

We extend the framework of dynamic Bayesian networks in order to incorporate perturbations. Moreover, an exact algorithm for inferring an optimal network is proposed and a discretization method specialized for time series data from perturbation experiments is introduced. We apply our procedure to realistic simulations data. The results are compared with those obtained by standard DBN learning techniques. Moreover, the advantages of using exact learning algorithm instead of heuristic methods are analyzed.

**Conclusion:**

We show that the quality of inferred networks dramatically improves when using data from perturbation experiments. We also conclude that the exact algorithm should be used when it is possible, i.e. when considered set of genes is small enough.

## Background

Since most genetic regulatory systems involve many components connected through complex networks of interactions, formal methods and computer tools for modeling and simulating are needed. Therefore, various formalisms were proposed to describe genetic regulatory systems, including Boolean networks and their generalizations, ordinary and partial differential equations, stochastic equations and Bayesian networks (see [1] for a review).

While differential and stochastic equations describe the biophysical processes at a very refined level of detail and prove useful in simulations of well studied systems, Bayesian networks appear attractive in the field of inferring the regulatory network structure from gene expression data [2]. The reason is that their learning techniques have a solid basis in statistics, allowing them to deal with the stochastic aspects of gene expressions and noisy measurements in a natural way. Other formalisms applied to this task include Boolean networks [3], weighted graphs [4], ordinary differential equations [5-7] and information-theoretic approaches [8].

A *Bayesian network *(BN) is a representation of a joint probability distribution over a set of random variables. It consists of two components:

• a directed acyclic graph whose vertices correspond to random variables and edges indicate conditional dependence relations

• a family of conditional distributions for each variable, given its parents in the graph.

Together, these two components determine a unique joint distribution.

When applying Bayesian networks to genetic regulatory systems, vertices are identified with genes and their expression levels, edges indicate interactions between genes and conditional distributions describe these interactions. Given a set of gene expression data, the learning techniques for Bayesian networks allow one to infer networks that match this set well. However, as it was shown in [9], the problem of finding an optimal network is NP-hard. Consequently, one has to choose between restricting to small gene networks (a relatively quick exponential algorithm was given in [10]) and inferring suboptimal networks by heuristic search methods (see [11]).

It should be also pointed out that the basic BN formalism has some major limitations. First, several networks with the same undirected graph structure but different directions of some edges may represent the same distribution. Hence, relying on expression levels only, the origin and the target of an interaction become indistinguishable. Second, the acyclicity constraint rules out feedback loops, essential in genetic networks. Third, the dynamics of a gene regulatory system is not taken into account.

The above limitations may be overcome by *Dynamic Bayesian networks *(DBNs), which model the stochastic evolution of a set of random variables over time. In comparison with BNs, discrete time is introduced and conditional distributions are related to the values of parent variables in the previous time point. Moreover, in DBNs the acyclicity constraint is relaxed.

Given a set of time series of expression data, the learning techniques adapted from BNs allow one to infer dynamic networks that match well the temporal evolution contained in the series. The papers [12] and [13] initiated a series of biological applications of DBNs [14-19].

A special treatment is required for experiments in which expression of some genes was perturbed (e.g. knockout experiments). Since perturbations change the structure of interactions (regulation of affected genes is excluded), the learning techniques have to use data selectively.

It should be also pointed out that not every perturbation experiment may be realized in practice. The reason is that some perturbations of a regulatory mechanism may be lethal to the organism. On the other hand data from perturbation experiments are particularly rich in information regarding causal relationships.

Inferring networks from perturbed expression profiles by means of BNs was investigated in [14] and [20]. To our knowledge the DBN technique has not been applied in the context of perturbation experiments. In the present paper we extend the framework of DBNs to deal with microarray data from perturbation experiments. We propose an exact algorithm for inferring an optimal network under BDe scoring function. Moreover, we propose a method of discretization of expression levels, suitable for the data coming from time series perturbation experiments. The above methodology is applied to realistic simulations data. We perform statistical analysis, via a suitably defined p-value, which assesses the statistical significance of the inferred networks. As a way of assessing the quality of the scoring function we estimate the percentage of networks with scores better than the score of the original network. We show that the quality of inferred networks dramatically improves when using data from perturbations. We also show some advantages of our exact algorithm over heuristics like Markov chain Monte Carlo (MCMC) method.

## Data and preprocessing

When analysing learning procedure's efficiency, the procedure should be applied to the data generated by a known network, which then might be compared with the inferred one. To this aim, most studies apply procedures to gene expression data and compare inferred interactions with those found in biological literature. The disadvantage of this approach is that our knowledge of the structures of real biological networks is far from being complete even in the most deeply investigated organisms. Although many interactions between genes are known, there are very few results stating the absence of some interactions. Thus no conclusion can be drawn from the fact that the procedure inferred unknown interaction. The above disadvantage is no longer present when data are generated from a mathematical model simulating real networks. However, a danger of this approach is that the employed model simplifies the real process, losing important biological features. In that case, analysis of a learning procedure is aimed at its ability to infer an artificial model rather than real biological networks.

Husmeier in [17] suggests that a satisfactory compromise between the above two extremes is to apply the learning procedure to data generated by a system of ordinary differential equations.

### Basic model

In the present study we generate data using the model proposed in [21]. The model consists of 54 species of molecules, representing 10 genes with their transcription factors, promoters, mRNAs, proteins and protein dimers, connected through 97 elementary reactions, including transcription factor binding, transcription, translation, dimerization, mRNA degradation and protein degradation. The dynamics of the model is described by the system of ordinary differential equations of the following form:

d[G2]dt=kdiG[G]2−kudG[G2]−kbiHG[G2][pH]+kubHG[G2⋅pH]−kdeG2[G2]d[pH]dt=−kbiHG[G2][pH]+kubHG[G2⋅pH]d[mH]dt=−kdmH[mH]+ktrH[pH]+ktrHG[G2⋅pH]d[H]dt=−kdeH[H]+ktlH[mH]
 MathType@MTEF@5@5@+=feaafiart1ev1aaatCvAUfKttLearuWrP9MDH5MBPbIqV92AaeXatLxBI9gBaebbnrfifHhDYfgasaacH8akY=wiFfYdH8Gipec8Eeeu0xXdbba9frFj0=OqFfea0dXdd9vqai=hGuQ8kuc9pgc9s8qqaq=dirpe0xb9q8qiLsFr0=vr0=vr0dc8meaabaqaciaacaGaaeqabaqabeGadaaakeaafaqabeabbaaaaeaadaWcaaqaaiabdsgaKjabcUfaBjabdEeahnaaBaaaleaacqaIYaGmaeqaaOGaeiyxa0fabaGaemizaqMaemiDaqhaaiabg2da9iabdUgaRnaaBaaaleaacqWGKbazcqWGPbqAcqWGhbWraeqaaOGaei4waSLaem4raCKaeiyxa01aaWbaaSqabeaacqaIYaGmaaGccqGHsislcqWGRbWAdaWgaaWcbaGaemyDauNaemizaqMaem4raCeabeaakiabcUfaBjabdEeahnaaBaaaleaacqaIYaGmaeqaaOGaeiyxa0LaeyOeI0Iaem4AaS2aaSbaaSqaaiabdkgaIjabdMgaPjabdIeaijabdEeahbqabaGccqGGBbWwcqWGhbWrdaWgaaWcbaGaeGOmaidabeaakiabc2faDjabcUfaBjabdchaWjabdIeaijabc2faDjabgUcaRiabdUgaRnaaBaaaleaacqWG1bqDcqWGIbGycqWGibascqWGhbWraeqaaOGaei4waSLaem4raC0aaSbaaSqaaiabikdaYaqabaGccqGHflY1cqWGWbaCcqWGibascqGGDbqxcqGHsislcqWGRbWAdaWgaaWcbaGaemizaqMaemyzauMaem4raC0aaSbaaWqaaiabikdaYaqabaaaleqaaOGaei4waSLaem4raC0aaSbaaSqaaiabikdaYaqabaGccqGGDbqxaeaadaWcaaqaaiabdsgaKjabcUfaBjabdchaWjabdIeaijabc2faDbqaaiabdsgaKjabdsha0baacqGH9aqpcqGHsislcqWGRbWAdaWgaaWcbaGaemOyaiMaemyAaKMaemisaGKaem4raCeabeaakiabcUfaBjabdEeahnaaBaaaleaacqaIYaGmaeqaaOGaeiyxa0Laei4waSLaemiCaaNaemisaGKaeiyxa0Laey4kaSIaem4AaS2aaSbaaSqaaiabdwha1jabdkgaIjabdIeaijabdEeahbqabaGccqGGBbWwcqWGhbWrdaWgaaWcbaGaeGOmaidabeaakiabgwSixlabdchaWjabdIeaijabc2faDbqaamaalaaabaGaemizaqMaei4waSLaemyBa0MaemisaGKaeiyxa0fabaGaemizaqMaemiDaqhaaiabg2da9iabgkHiTiabdUgaRnaaBaaaleaacqWGKbazcqWGTbqBcqWGibasaeqaaOGaei4waSLaemyBa0MaemisaGKaeiyxa0Laey4kaSIaem4AaS2aaSbaaSqaaiabdsha0jabdkhaYjabdIeaibqabaGccqGGBbWwcqWGWbaCcqWGibascqGGDbqxcqGHRaWkcqWGRbWAdaWgaaWcbaGaemiDaqNaemOCaiNaemisaGKaem4raCeabeaakiabcUfaBjabdEeahnaaBaaaleaacqaIYaGmaeqaaOGaeyyXICTaemiCaaNaemisaGKaeiyxa0fabaWaaSaaaeaacqWGKbazcqGGBbWwcqWGibascqGGDbqxaeaacqWGKbazcqWG0baDaaGaeyypa0JaeyOeI0Iaem4AaS2aaSbaaSqaaiabdsgaKjabdwgaLjabdIeaibqabaGccqGGBbWwcqWGibascqGGDbqxcqGHRaWkcqWGRbWAdaWgaaWcbaGaemiDaqNaemiBaWMaemisaGeabeaakiabcUfaBjabd2gaTjabdIeaijabc2faDbaaaaa@F62C@

where *t *represents time, *k*_*x *_are kinetic constants of related reactions, [*X*] means concentration, *pX*, *mX*, *X *and *X*_2 _are promoter, mRNA, protein and dimer *X*, respectively, finally *X*·*Y *stands for a transcription factor bound to a promoter.

**Figure 1:**
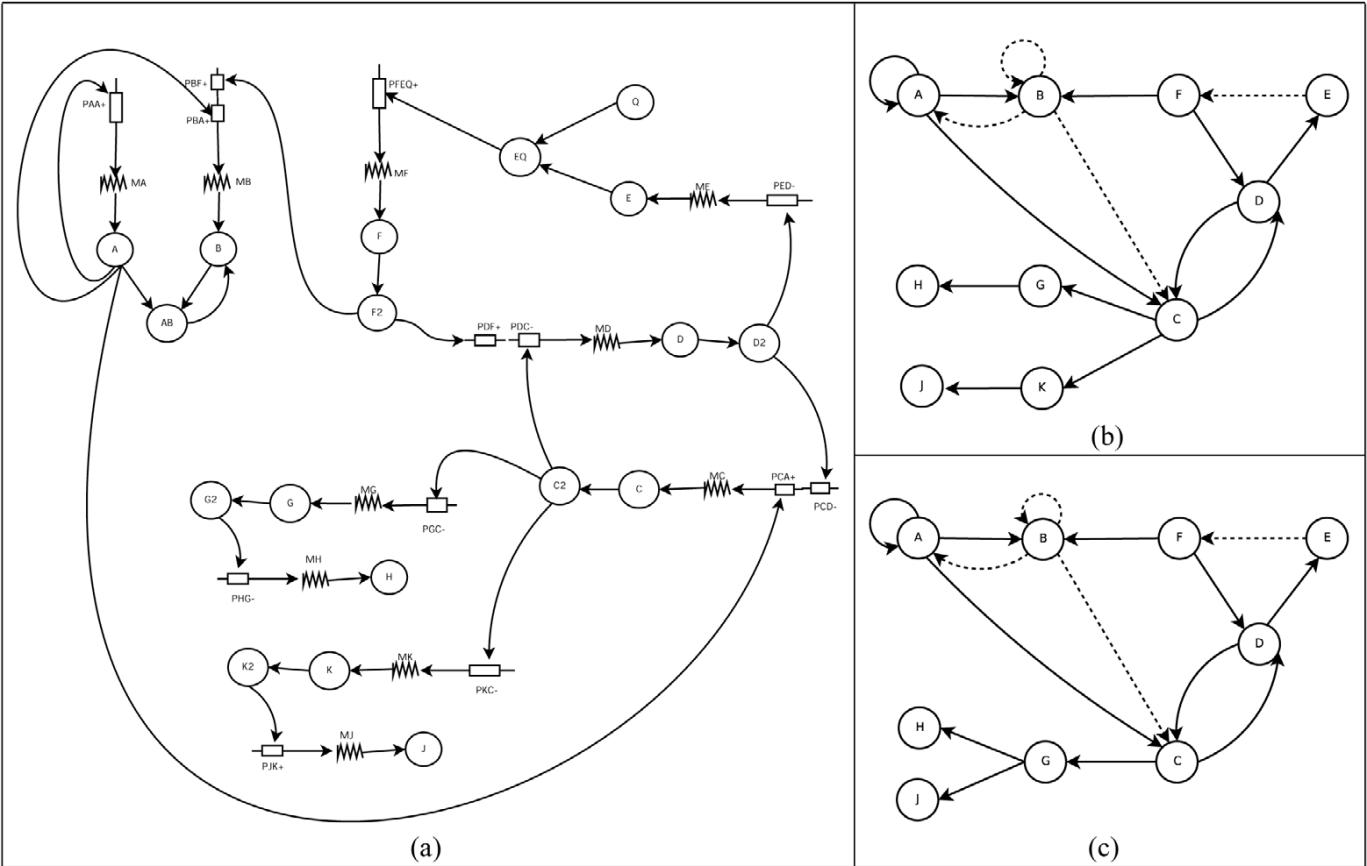
**The gene regulatory network from [21], its mRNA interactions and the corresponding diagram for the network modified by [17]**. (a) The gene regulatory network from [21]. Rectangles denote promoters, zigzags indicate mRNAs and circles stand for proteins, dimers and a ligand (Q). The symbols + and - indicate whether transcription of a gene is activated or inhibited by relevant transcription factor. The subnetwork involving the genes *A *and *B *is a hysteretic oscillator [22]. Protein *A *activates transcription of both genes. Protein *B *joins it forming a dimer *AB*, which reduces the amount of free protein *A *and, consequently, inhibits transcription. Thus oscillations appear. The genes *C *and *D *compose a switch [23]: each protein forms a dimer which acts as an inhibitor of transcription of the other gene. Therefore highly expressed gene switches off expression of the other. The ligand binding mechanism [24] is represented by the subnetwork involving the genes *E *and *F*: protein *E *joined with a ligand *Q *forms an activator of transcription of the gene *F*. Finally, there are two cascades: in the first cascade *C *inhibits *G *and *G *inhibits *H*, while in the second cascade *C *inhibits *K *and *K *activates *J*. (b) The mRNA interactions in the above network; solid arrows denote transcriptional regulation, dashed ones represent interactions triggered by the ligand and posttransciptional regulation, (c) The corresponding diagram for the network modified by [17].

The system is composed of structures reported in the biological literature [22-24], i.e. a hysteretic oscillator, a genetic switch, cascades and a ligand binding mechanism that influences transcription (during the simulation, the ligand is injected for a short time). The whole network is shown in Fig. 1(a).

The total time of each simulation is set to 5000 minutes. At time 1000 minutes the ligand is injected for 10 minutes, changing the expression levels of all genes. The influence of the injection to expression decays throughout the interval 1500–3200 minutes (depending on the gene), but at time 2400 minutes system dynamics becomes similar to the initial one.

All the equations and parameters of the model, as well as the MATLAB code to integrate it, are available in the supplementary materials to [21].

This model is chosen for two reasons. First, differential equations formalism and biological origin of the structure guarantee realistic simulations. Second, small size of the system (note that, according to microarray experiments data, the learning procedure will be provided with mRNA concentrations only) allows to avoid a noise arising from heuristic search methods, which are necessary when dealing with large networks. Such noise might influence comparison of methods.

### Modified models

Since genes *G *and *K *from the model are regulated by the same gene *C *and have the same kinetic constants, trajectories of their concentrations are identical. Consequently, their contributions to the regulatory interactions are indistinguishable, given the expression data. For this reason, Husmeier in [17] tests efficiency of DBN based learning techniques using the model slightly modified by identifying both genes.

In the present study we introduce perturbations to the model. It is done by replacing the differential equation regarding the mRNA of a perturbed gene by the following equation:

d[mX]dt=kpeX(c−[mX])
 MathType@MTEF@5@5@+=feaafiart1ev1aaatCvAUfKttLearuWrP9MDH5MBPbIqV92AaeXatLxBI9gBaebbnrfifHhDYfgasaacH8akY=wiFfYdH8Gipec8Eeeu0xXdbba9frFj0=OqFfea0dXdd9vqai=hGuQ8kuc9pgc9s8qqaq=dirpe0xb9q8qiLsFr0=vr0=vr0dc8meaabaqaciaacaGaaeqabaqabeGadaaakeaadaWcaaqaaiabdsgaKjabcUfaBjabd2gaTjabdIfayjabc2faDbqaaiabdsgaKjabdsha0baacqGH9aqpcqWGRbWAdaWgaaWcbaGaemiCaaNaemyzauMaemiwaGfabeaakiabcIcaOiabdogaJjabgkHiTiabcUfaBjabd2gaTjabdIfayjabc2faDjabcMcaPaaa@4585@

which makes the concentration exponentially converging to *c*. Taking *c *= 0 yields system with gene knocked out, while setting *c *to maximal (with respect to the basic system) expression level of a gene makes it overexpressed. 21 simulations altogether are executed: one simulation with the basic system and 20 simulations with one gene knocked out or overexpressed.

Octave scripts for generating expression time series are available in the supplementary materials [25].

### Sampling and discretization

Husmeier in [17] chooses for his test 12 time points in equal length intervals between 1100 and 1600 minutes. He argues that more information is contained in the data derived from the system which is driven out of equilibrium by the ligand injection. In our tests Husmeier's choice is repeated and other intervals are tried, as reported below.

Before applying our learning procedure (as well as Husmeier's), the expression levels need to be discretize. One of the simplest methods of discretizing is partition of the interval of real numbers covering mRNAs concentrations of each gene into subintervals of equal length, relevant to particular discretized values. Another strategy is to base the discretization procedure on the meanings of introduced discrete expression levels (e.g. 'on'-'off' or 'under-expressed'-'baseline'-'over-expressed').

Husmeier in [17] applies the former approach with 3 discretized expression levels, and we follow him in the case of unperturbed data.

**Figure 2:**
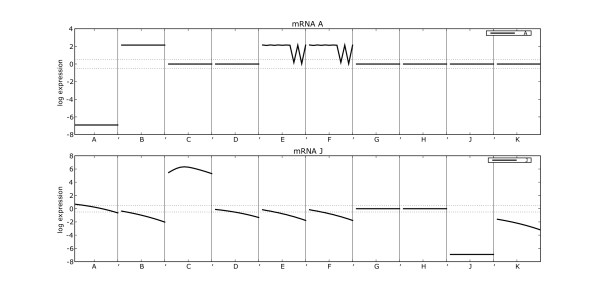
**Log-ratios of mRNA concentration values for all knockout experiments**. Log-ratios of mRNA concentration values of genes *A *and *J *for all knockout experiments. 12 time points in equal length intervals between 1100 and 1600 minutes are taken from each experiment (120 slices together). Ratios lower than 0.001 were set to this value. The horizontal lines indicate the top and bottom limits of the baseline expression level. Extended version of the figure including all 10 genes is available in the supplementary materials [25].

The specificity of perturbed data suggests the latter approach. The simulation of an unperturbed system specifies the reference point for expression levels of perturbed data. Thus discretization consists in comparing each concentration value from a perturbed system simulation with the concentration value of the same gene at the same time point of the unperturbed system simulation. When the values are close to each other, i.e.



the expression level is set to 'baseline', otherwise it is set to 'over-' or 'under-expressed'. The log-ratios of concentration values in knockout simulations are shown in Fig. 2. The threshold 0.5 seems to minimize the loss of information, inevitable in the discretization process. However, this choice, as well as the choice of the number of thresholds, is arbitrary.

Discretized expression data files are available in the supplementary materials [25].

## Methods

### Dynamic Bayesian networks

A *dynamic Bayesian network *N
 MathType@MTEF@5@5@+=feaafiart1ev1aaatCvAUfKttLearuWrP9MDH5MBPbIqV92AaeXatLxBI9gBamrtHrhAL1wy0L2yHvtyaeHbnfgDOvwBHrxAJfwnaebbnrfifHhDYfgasaacH8akY=wiFfYdH8Gipec8Eeeu0xXdbba9frFj0=OqFfea0dXdd9vqai=hGuQ8kuc9pgc9s8qqaq=dirpe0xb9q8qiLsFr0=vr0=vr0dc8meaabaqaciaacaGaaeqabaWaaeGaeaaakeaaimaacqWFneVtaaa@383B@ is a representation of stochastic evolution of a set of random variables **X **= {*X*_1_,..., *X*_*n*_} over discretized time. Represented temporal process is assumed to be *Markovian*, i.e.

*P*(**X**(*t*)|**X**(0), **X**(1),..., **X**(*t *- 1)) = *P*(**X**(*t*)|**X**(*t *- 1))

and *time homogenous*, i.e.*P*(**X**(*t*)|**X**(*t *- 1)) are independent of *t*. The representation consists of two components:

• a directed graph *G *= (**X**, **E**) encoding con ditional (in-)dependencies

• a family of conditional distributions *P*(*X*_*i*_(*t*)|**Pa**_*i*_(*t *- 1)), where **Pa**_*i *_= {*X*_*j *_∈ **X**|(*X*_*j*_, *X*_*i*_) ∈ **E**}

By assumption, the joint distribution over all the possible trajectories of the process decomposes into the following product form

P(X(0),X(1),…,X(T))=P(X(0))∏t=1TP(X(t)|X(t−1))
 MathType@MTEF@5@5@+=feaafiart1ev1aaatCvAUfKttLearuWrP9MDH5MBPbIqV92AaeXatLxBI9gBaebbnrfifHhDYfgasaacH8akY=wiFfYdH8Gipec8Eeeu0xXdbba9frFj0=OqFfea0dXdd9vqai=hGuQ8kuc9pgc9s8qqaq=dirpe0xb9q8qiLsFr0=vr0=vr0dc8meaabaqaciaacaGaaeqabaqabeGadaaakeaacqWGqbaucqGGOaakieqacqWFybawcqGGOaakcqaIWaamcqGGPaqkcqGGSaalcqWFybawcqGGOaakcqaIXaqmcqGGPaqkcqGGSaalcqWIMaYscqGGSaalcqWFybawcqGGOaakcqWGubavcqGGPaqkcqGGPaqkcqGH9aqpcqWGqbaucqGGOaakcqWFybawcqGGOaakcqaIWaamcqGGPaqkcqGGPaqkdaqeWbqaaiabdcfaqjabcIcaOiab=HfayjabcIcaOiabdsha0jabcMcaPiabcYha8jab=HfayjabcIcaOiabdsha0jabgkHiTiabigdaXiabcMcaPiabcMcaPaWcbaGaemiDaqNaeyypa0JaeGymaedabaGaemivaqfaniabg+Givdaaaa@5C7E@

Consequently, the evolution of the random variables is given by

P(X(1),…,X(T)|X(0))=∏t=1TP(X(t)|X(t−1))=∏t=1T∏i=1nP(Xi(t)|Pai(t−1))==∏i=1n∏t=1TP(Xi(t)|Pai(t−1))     (1)
 MathType@MTEF@5@5@+=feaafiart1ev1aaatCvAUfKttLearuWrP9MDH5MBPbIqV92AaeXatLxBI9gBaebbnrfifHhDYfgasaacH8akY=wiFfYdH8Gipec8Eeeu0xXdbba9frFj0=OqFfea0dXdd9vqai=hGuQ8kuc9pgc9s8qqaq=dirpe0xb9q8qiLsFr0=vr0=vr0dc8meaabaqaciaacaGaaeqabaqabeGadaaakeaafaqaceGabaaabaGaemiuaaLaeiikaGccbeGae8hwaGLaeiikaGIaeGymaeJaeiykaKIaeiilaWIaeSOjGSKaeiilaWIae8hwaGLaeiikaGIaemivaqLaeiykaKIaeiiFaWNae8hwaGLaeiikaGIaeGimaaJaeiykaKIaeiykaKIaeyypa0ZaaebCaeaacqWGqbaucqGGOaakcqWFybawcqGGOaakcqWG0baDcqGGPaqkcqGG8baFcqWFybawcqGGOaakcqWG0baDcqGHsislcqaIXaqmcqGGPaqkcqGGPaqkcqGH9aqpdaqeWbqaamaarahabaGaemiuaaLaeiikaGIaemiwaG1aaSbaaSqaaiabdMgaPbqabaGccqGGOaakcqWG0baDcqGGPaqkcqGG8baFcqWFqbaucqWFHbqydaWgaaWcbaGaemyAaKgabeaakiabcIcaOiabdsha0jabgkHiTiabigdaXiabcMcaPiabcMcaPiabg2da9aWcbaGaemyAaKMaeyypa0JaeGymaedabaGaemOBa4ganiabg+GivdaaleaacqWG0baDcqGH9aqpcqaIXaqmaeaacqWGubava0Gaey4dIunaaSqaaiabdsha0jabg2da9iabigdaXaqaaiabdsfaubqdcqGHpis1aaGcbaGaeyypa0ZaaebCaeaadaqeWbqaaiabdcfaqjabcIcaOiabdIfaynaaBaaaleaacqWGPbqAaeqaaOGaeiikaGIaemiDaqNaeiykaKIaeiiFaWNae8huaaLae8xyae2aaSbaaSqaaiabdMgaPbqabaGccqGGOaakcqWG0baDcqGHsislcqaIXaqmcqGGPaqkcqGGPaqkaSqaaiabdsha0jabg2da9iabigdaXaqaaiabdsfaubqdcqGHpis1aaWcbaGaemyAaKMaeyypa0JaeGymaedabaGaemOBa4ganiabg+GivdaaaOGaaCzcaiaaxMaadaqadaqaaiabigdaXaGaayjkaiaawMcaaaaa@9F49@

### Inferring networks

The problem of learning a DBN is understood as follows: find a network graph that best matches a given dataset of time series of **X**-instances. The notion of a good match is formalized by means of a *scoring function*, usually Bayesian Dirichlet equivalence (BDe) score [12,26], derived from the posterior probability of the network, given the data (the prior distributions over the network parameters have to be assumed). Owing to the product decomposition (1), BDe score decomposes into the sum over the set of random variables. This property is extremely useful in learning procedures, since the parents of each variable may be computed independently. When the set of variables is small enough (the boundary is approximately 20), one may score each subset as a possible parent set for each variable and choose the best match. Otherwise, heuristic search methods have to be applied and the decomposition property is helpful in reducing the computation cost when scoring locally changed networks.

Since our training datasets consist of mRNA concentrations of 10 genes only, we can apply an exact algorithm. This choice allows us to avoid the noise caused by using heuristic search methods.

Edges in the inferred network graph witness conditional dependence between variables in neighboring time points, which is interpreted as interaction between corresponding genes. However, a special care is required when inferring self-regulation. In this case it is clear that *X*_*i*_(*t *+ 1) depends on *X*_*i*_(*t*) because of natural inertia of mRNA production and degradation. Such dependence cannot be distinguished from actual auto-regulation by the scoring function currently used to select the best DBN model for the data. In the particular case of our experiments we have observed that with different choice of the number of time points we obtained all or none of the genes with self loops. This issue was addressed in other studies [12,15] by explicitly forbidding or forcing the presence of self-loops in all considered models. We take the same approach in the present paper. However it remains an open question whether the DBN scoring functions could be extended to distinguish between inertia and self-dependence.

### Perturbations

When expression of a gene is perturbed in an experiment (e.g. by knocking it out), its natural regulation is blocked and replaced by the perturbation scheme. Consequently, regarding that gene's regulation mechanisms, the experiment contributes noise to the model instead of information. On the other hand, the remaining interactions might be significantly reflected in data, in particular those in which the gene acts as a regulator. Therefore our learning procedure has to make use of data from perturbation experiments selectively.

Recall that the parent sets of each gene may be inferred independently. Thus, when inferring parents of a particular gene, we apply the standard learning procedure to the dataset restricted to those experiments, in which this gene's expression was not perturbed. When parents of all genes are computed, the network graph is composed. A related method in the framework of static BNs was successfully used in [14] and [20]. Summarizing, our exact algorithm can be expressed as follows:

for each gene **G**

   choose all experiments with unperturbed expression of **G**

   for each potential parent set Pa of **G**

      compute the local score in **G** for **Pa** and chosen experiments

   choose the parent set of **G** yielding optimal score compose the network from the chosen parent sets

Software for finding optimal DBNs is available in the supplementary materials [25].

## Results and discussion

In the present section our exact algorithm is applied to the datasets from the model modified by introducing perturbations. The results are compared with those obtained from the basic model, as well as with those obtained by Bayesian learning with Markov chain Monte Carlo (MCMC) method [17].

### Experiments

**Figure 3:**
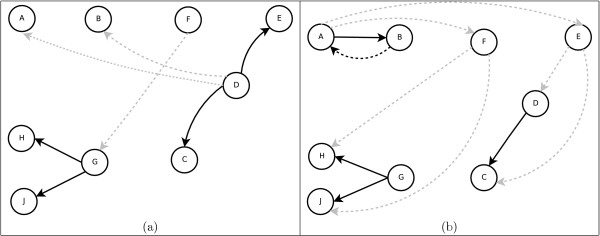
**The interactions inferred from unperturbed data**. The interactions inferred from unperturbed data (12 slices; the network restricted to 9 genes): (a) by our exact algorithm with self-loops forbidden (the edges occurring in each network with the optimal score) and (b) by Markov chain Monte Carlo method [17]; black arrows show true inferred edges (solid arrows refer to transcriptional regulation and dashed refer to interactions triggered by the ligand and posttranscriptional regulation), grey dashed arrows represent spurious edges.

In the first experiment we followed the procedure of Husmeier [17] (the system restricted to 9 genes, 12 time points chosen in equal length intervals between 1100 and 1600 minutes, simple discretization into 3 levels). The sensitivity of our exact algorithm was similar to Husmeier's heuristics – see Fig. 3.

Next we turned to the knockout data. Recall that the entire system with all 10 genes was considered and the discretization was made according to the comparison of expression levels from perturbed and unperturbed profiles.

**Figure 4:**
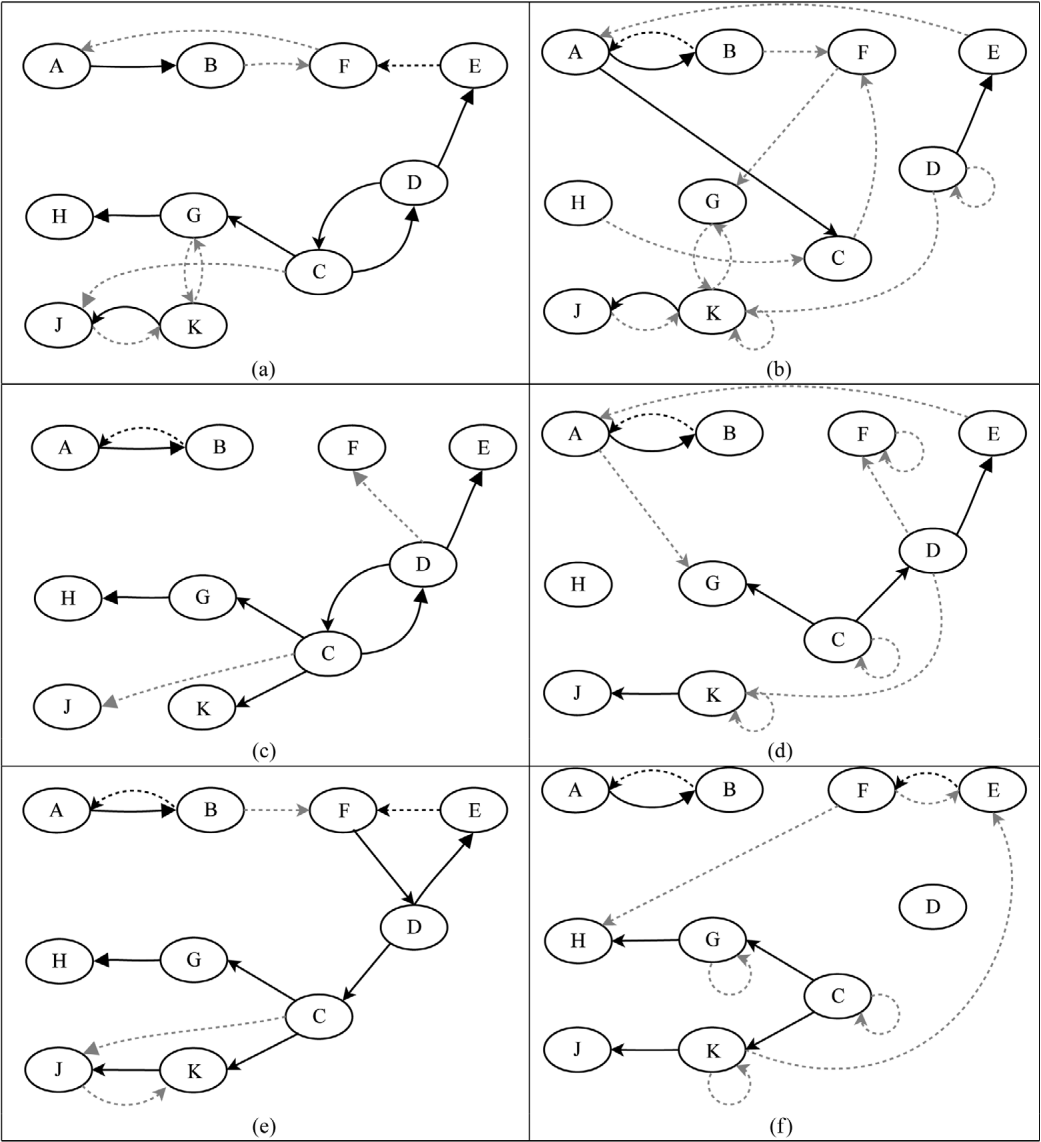
**The interactions inferred from perturbed data**. The interactions inferred from perturbed data: (ab) 10 knockout series, each with 12 slices, (cd) 10 knockout series, each with 3 slices, (ef) 10 knockout and 10 overexpression series, each with 3 slices; black arrows show true inferred edges (solid arrows refer to transcriptional regulation and dashed refer to interactions triggered by the ligand and posttranscriptional regulation), grey dashed arrows represent spurious edges. The networks (ace) are obtained by our exact algorithm with self-loops forbidden (if there are many networks with the optimal score, the edges occurring in each one are shown) and the networks (bdf) by the MCMC method [17].

The first set of time points was chosen as in the above experiment, resulting in 7 edges corresponding to direct transcriptional regulation, 1 edge due to an interaction triggered by the ligand and 6 spurious edges (see Fig. 4(a)).

The dataset used in the experiment was quite large: 10 series, 12 time points each gives 120 slices. On the other hand, the variability of discretized expression levels is rather low – as is shown in Fig. 2, the thresholds are usually crossed at most one time per series. Therefore the steadiness of expression is represented in enormous proportion. Moreover, the sampling rate fails to match the delay of regulation processes. Since edges in DBNs represent conditional dependencies in neighboring time points, the learning process is affected. Consequently, a large number of false positives appears in the inferred network. The variability of expression is reduced by the discretization process. The choice of our discretization threshold 0.5 is aimed at minimizing this reduction (see the section *Sampling and discretization*). The variability may be increased by allowing more discretization levels, but it can disturb inferring. The reason is that the BN formalism disregards the structure of sets of possible values of random variables. For example, the information that the discretized expression level '0' is closer to the level '1' than to '2' is ignored. Consequently, the learning procedure treats equally the situation in which some configuration of regulators causes a regulon to assume the value '0' or '1' with the one in which it is caused to assume the value '0' or '2'. Our experiments with gene expression discretized into more than 3 levels do not improve results (data not shown).

The disproportion between a large dataset and a low variability may be avoided by decreasing a number of samples. Hence we decided to choose for the next experiment 3 time points in equal length intervals between 1100 and 1600 minutes. The accuracy significantly improved – the inferred network contains 7 edges corresponding to direct transcriptional regulation, 1 reflecting posttranscriptional regulation and 2 spurious edges (see Fig. 4(c)).

Another time intervals were tried, resulting in networks less accurate than the two above (data not shown), which confirms Husmeier's assertion of low information content of signals from a system being in equilibrium.

Corresponding experiments were also executed for the overexpression data, as well as for both kinds of perturbed data together. Accuracy of overexpression experiments does not match that of knockout ones. However, it is worth pointing out that, unlike the knockout data case, the edges *A*→*C*, *B*→*C *and *E*→*F *were inferred correctly.

The best results were obtained when both kinds of perturbations were used together. As it is shown on Fig. 4(e), the inferred network contains 8 edges corresponding to direct transcriptional regulation, 1 edge due to an interaction triggered by the ligand, 1 reflecting posttranscriptional regulation and 3 spurious edges. The last experiment aimed at comparing our exact algorithm with heuristic methods of searching networks with optimal scores. We adapted the MCMC algorithm of Husmeier [17] to work with perturbations and applied it to our data. The accuracy of obtained networks was lower then the one of networks resulting from our exact algorithm – see Fig. 4. Moreover, the experiments showed two disadvantages of this method. First, when the number of genes is small (10 genes in the considered network), the MCMC algorithm is significantly slower than the exact one. Second, due to the non-deterministic character of the algorithm, the networks inferred in succeeding simulations were highly variable. For example, Husmeier's experiment on unperturbed data, repeated 100 times, resulted in the set of networks with the number of correctly inferred edges varying from 1 to 5. Moreover, the network obtained by Husmeier (see Fig. 3(b)) did not appear among them.

### Statistical analysis

We define the *p-value *of a network with *k *true out of *m *inferred edges to be the probability of finding at least *k *true when choosing *m *edges at random. According to the hypergeometric distribution, the probability of *n *successful selections out of *m *from a set of *N *true and *M *- *N *false edges is given by

pn=(Nn)(M−Nm−n)(Mm)
 MathType@MTEF@5@5@+=feaafiart1ev1aaatCvAUfKttLearuWrP9MDH5MBPbIqV92AaeXatLxBI9gBaebbnrfifHhDYfgasaacH8akY=wiFfYdH8Gipec8Eeeu0xXdbba9frFj0=OqFfea0dXdd9vqai=hGuQ8kuc9pgc9s8qqaq=dirpe0xb9q8qiLsFr0=vr0=vr0dc8meaabaqaciaacaGaaeqabaqabeGadaaakeaacqWGWbaCdaWgaaWcbaGaemOBa4gabeaakiabg2da9maalaaabaWaaeWaaeaafaqabeGabaaabaGaemOta4eabaGaemOBa4gaaaGaayjkaiaawMcaamaabmaabaqbaeqabiqaaaqaaiabd2eanjabgkHiTiabd6eaobqaaiabd2gaTjabgkHiTiabd6gaUbaaaiaawIcacaGLPaaaaeaadaqadaqaauaabeqaceaaaeaacqWGnbqtaeaacqWGTbqBaaaacaGLOaGaayzkaaaaaaaa@4182@

consequently,the p-value of a network is defined by

pval=∑n=kmin(m,N)(Nn)(M−Nm−n)(Mm)
 MathType@MTEF@5@5@+=feaafiart1ev1aaatCvAUfKttLearuWrP9MDH5MBPbIqV92AaeXatLxBI9gBaebbnrfifHhDYfgasaacH8akY=wiFfYdH8Gipec8Eeeu0xXdbba9frFj0=OqFfea0dXdd9vqai=hGuQ8kuc9pgc9s8qqaq=dirpe0xb9q8qiLsFr0=vr0=vr0dc8meaabaqaciaacaGaaeqabaqabeGadaaakeaacqWGWbaCcqWG2bGDcqWGHbqycqWGSbaBcqGH9aqpdaaeWbqaamaalaaabaWaaeWaaeaafaqabeGabaaabaGaemOta4eabaGaemOBa4gaaaGaayjkaiaawMcaamaabmaabaqbaeqabiqaaaqaaiabd2eanjabgkHiTiabd6eaobqaaiabd2gaTjabgkHiTiabd6gaUbaaaiaawIcacaGLPaaaaeaadaqadaqaauaabeqaceaaaeaacqWGnbqtaeaacqWGTbqBaaaacaGLOaGaayzkaaaaaaWcbaGaemOBa4Maeyypa0Jaem4AaSgabaacbiGae8xBa0Mae8xAaKMae8NBa4MaeiikaGIaemyBa0MaeiilaWIaemOta4KaeiykaKcaniabggHiLdaaaa@5350@

where *M *equals 10^2 ^for the full considered network or 9^2 ^for the network restricted to 9 genes (used for unperturbed data). The value of *N *depends on it if we allow direct transcriptional regulation only. P-values of the networks from Fig. 3 and 4 are grouped in the Table 1.

The above considerations refer to inferring *local *interactions between genes, represented by particular edges. In order to analyse the ability to infer a *global *interaction system, one has to compare the score of the original network with the scores of other networks. Since it is impossible to compute the scores of all graphs (there are 2n2
 MathType@MTEF@5@5@+=feaafiart1ev1aaatCvAUfKttLearuWrP9MDH5MBPbIqV92AaeXatLxBI9gBaebbnrfifHhDYfgasaacH8akY=wiFfYdH8Gipec8Eeeu0xXdbba9frFj0=OqFfea0dXdd9vqai=hGuQ8kuc9pgc9s8qqaq=dirpe0xb9q8qiLsFr0=vr0=vr0dc8meaabaqaciaacaGaaeqabaqabeGadaaakeaacqaIYaGmdaahaaWcbeqaaiabd6gaUnaaCaaameqabaGaeGOmaidaaaaaaaa@3050@ directed graph structures with *n *nodes), we sampled randomly 100 000 graphs. For each graph, edges were generated independently, each with the same probability. The uniform distribution on the space of all graphs could be obtained by setting this probability to 1/2, but it would cause scores of most of graphs to be dominated by high penalties due to excessive structures. In order to get networks with scores close to the original one, there was chosen the probability resulting in the expected number of 12 edges in the graph (11 edges between different nodes in the cases of forbidden and forced self-connecting edges).

Original and randomly generated graphs are available in the supplementary materials [25].

Table 2 shows how many generated networks received (in various experiments) score better then the original one. We compare the results obtained with forbidden, permitted or forced self-loops, i.e. edges leading from a vertex to itself.

The tables show that using perturbed data significantly improves the possibility of inferring the original network. The results obtained in the experiments with 3 time points are usually better than those in the experiment with 12 time points, but the differences between them are relatively small.

Comparison of the values for particular versions of the algorithm shows that the best results are obtained when self-loops are forbidden, slightly worse when self-loops are permitted and significantly worse when they are forced. The analysis of the best scored networks with permitted self-loops leads to the statement that self-regulation of genes cannot be handled within our framework correctly and requires more refined methods. Therefore, with respect to our algorithm's variants, the best choice is to forbid self-loops.

## Conclusion

In the present paper the framework of dynamic Bayesian networks is extended in order to handle gene expression perturbations. A new discretization method specialized for datasets from time series gene perturbation experiments is also introduced. Networks inferred from realistic simulations data by our method are compared with those obtained by DBNs learning techniques.

The comparison shows that application of our method substantially improves quality of inference. Moreover, our results lead to the suggestion that the exact algorithm should be applied when it is possible, i.e. when the set of genes is small enough. The reason is high variability of the networks resulting from heuristics and their lower accuracy.

Since self-regulating interactions appeared to be intractable by DBN learning techniques, we also suggest to forbid self-connecting edges in inferred networks. Our experiments show that this choice makes the learning procedure more sensitive to other interactions than it would be with self-loops permitted or forced. An important problem for designing time series expression experiments is to determine sampling rates properly. Our experiments show that assuming too short rate results in noisy expression profiles, just as when the samples are chosen from the system being in equilibrium. Consequently, networks inferred from over-sampled datasets have low accuracy.

The reason of this surprising finding is the Markovian assumption of DBNs, which states that the value of an expression profile from a particular time point depends on the value of the profile from the preceding time point only. It means that the sampling rate has to match the delay of regulation processes. Most learning procedures working with time series gene expression data make similar assumptions. This is unlike those working with independent expression profiles (e.g. BNs), which assume that considered profiles represent steady states.

## Authors' contributions

ND designed the extension of DBN framework incorporating perturbations, performed the statistical analysis and participated in executing the experiments. AG participated in the design of the study. AM implemented and tested the exact algorithm. BW implemented modifications concerning perturbations: to the system of differential equations of regulatory network [21] and to the MCMC learning procedure [17] and participated in executing the experiments. JT coordinated the study. ND, BW and JT edited the final manuscript. All authors read and approved the final manuscript.

## Figures and Tables

**Table 1:** The p-values of inferred networks.

dataset		all regulatory interactions		transcriptional regulation only	
perturbations	time points	exact	MCMC	exact	MCMC
-	12	0.0199	0.0266	0.0055	0.0381
knockout	12	3.4·10^-5^	0.0803	5.4·10^-5^	0.0978
knockout	3	6.4·10^-7^	0.0058	2.2·10^-6^	0.0080
both	3	1.4·10^-8^	0.0007	9.8·10^-7^	0.0080

**Table 2:** The percentages of generated networks with scores better than one of the original network. In the options of the algorithm with forbidden or forced self-loops, the original network was modified by removing or adding appropriate edges, respectively.

dataset		all regulatory interactions			transcriptional regulation only		
perturbations	time points	s-l forb.	s-l perm.	s-l forc.	s-l forb.	s-l perm.	s-l forc.
-	12	12.642	24.309	53.245	02.925	02.876	20.842
knockout	12	00.000	00.009	03.901	00.015	00.119	05.328
knockout	3	00.000	00.001	08.661	00.001	00.000	04.330
both	3	00.000	00.003	09.642	00.000	00.005	04.618
